# Novel approach to continuous adventitious respiratory sound analysis for the assessment of bronchodilator response

**DOI:** 10.1371/journal.pone.0171455

**Published:** 2017-02-08

**Authors:** Manuel Lozano-García, José Antonio Fiz, Carlos Martínez-Rivera, Aurora Torrents, Juan Ruiz-Manzano, Raimon Jané

**Affiliations:** 1 Biomedical Signal Processing and Interpretation Group, Institute for Bioengineering of Catalonia (IBEC), Barcelona, Spain; 2 Biomedical Research Networking Centre in Bioengineering, Biomaterials and Nanomedicine (CIBER-BBN), Barcelona, Spain; 3 Pulmonology Service, Germans Trias i Pujol University Hospital, Badalona, Spain; 4 Department of Automatic Control (ESAII), Universitat Politècnica de Catalunya (UPC)-Barcelona Tech, Barcelona, Spain; Universitatsklinikum Freiburg, GERMANY

## Abstract

**Background:**

A thorough analysis of continuous adventitious sounds (CAS) can provide distinct and complementary information about bronchodilator response (BDR), beyond that provided by spirometry. Nevertheless, previous approaches to CAS analysis were limited by certain methodology issues. The aim of this study is to propose a new integrated approach to CAS analysis that contributes to improving the assessment of BDR in clinical practice for asthma patients.

**Methods:**

Respiratory sounds and flow were recorded in 25 subjects, including 7 asthma patients with positive BDR (BDR+), assessed by spirometry, 13 asthma patients with negative BDR (BDR-), and 5 controls. A total of 5149 acoustic components were characterized using the Hilbert spectrum, and used to train and validate a support vector machine classifier, which distinguished acoustic components corresponding to CAS from those corresponding to other sounds. Once the method was validated, BDR was assessed in all participants by CAS analysis, and compared to BDR assessed by spirometry.

**Results:**

BDR+ patients had a homogenous high change in the number of CAS after bronchodilation, which agreed with the positive BDR by spirometry, indicating high reversibility of airway obstruction. Nevertheless, we also found an appreciable change in the number of CAS in many BDR- patients, revealing alterations in airway obstruction that were not detected by spirometry. We propose a categorization for the change in the number of CAS, which allowed us to stratify BDR- patients into three consistent groups. From the 13 BDR- patients, 6 had a high response, similar to BDR+ patients, 4 had a noteworthy medium response, and 1 had a low response.

**Conclusions:**

In this study, a new non-invasive and integrated approach to CAS analysis is proposed as a high-sensitive tool for assessing BDR in terms of acoustic parameters which, together with spirometry parameters, contribute to improving the stratification of BDR levels in patients with obstructive pulmonary diseases.

## Introduction

Asthma is a chronic respiratory disease characterized by airway inflammation and the resulting airway obstruction. The clinical features of asthma include variable respiratory symptoms, such as wheezes, and variable airflow limitation [1]. Airflow limitation is commonly assessed by spirometry. The most reliable parameters in spirometry are the forced expiratory volume in 1 second (FEV_1_) and its ratio to forced vital capacity (FVC). A reduced FEV_1_, when accompanied by a decrease in the FEV_1_/FVC ratio below the normal range of values (0.75–0.8), is a clear indicator of airway obstruction and airflow limitation [1].

When airway obstruction is confirmed from spirometry parameters, a bronchodilator response (BDR) test is usually performed to measure variation in airflow limitation. The BDR test consists of measuring the improvement in FEV_1_ within minutes after inhalation of a short-acting beta_2_-agonist bronchodilator [2]. Bronchodilators cause the airway smooth muscles to relax, which allows the airways to dilate, thus reducing airflow limitation. Obtaining evidence of high variability in airflow limitation is one of the main components of asthma diagnosis. Moreover, assessing BDR periodically after the initial diagnosis of asthma is an essential part of asthma control, since uncontrolled asthma is associated with a greater BDR than well-controlled asthma [1].

Despite the widespread use of the BDR test, the standard BDR criterion is still a subject of controversy [3–5]. The percentage increase in FEV_1_ is highly influenced by the baseline FEV_1_ [1]. Subjects with a low baseline FEV_1_ are more likely to have a greater BDR than subjects with a high baseline FEV_1_, who hardly ever have a positive BDR [6]. Therefore, a diagnosis of asthma should not be made based only on spirometry parameters [2].

The assessment of respiratory symptoms is also a major component of asthma diagnosis and control [1, 7]. Together with lung function, asthma symptoms should be assessed as often as possible. The common procedures for assessing asthma symptoms include physical examination and direct questioning, and the most frequent finding is the presence of wheezes—the most common type of continuous adventitious sounds (CAS)—on auscultation [1]. CAS are musical sounds with a sinusoidal-like waveform and a duration of over 100 ms [8]. In particular, wheezes have a pitch between 100 Hz and 1000 Hz and are generated by oscillation of airway walls in narrowed airways, as in asthma. Besides wheezes, CAS include rhonchi, which are similar to wheezes but coarser and lower in pitch (around 150 Hz), are often related to the movement of air through secretions, and have a gurgling or snoring-like quality [8, 9]. However, the term rhonchi is often used to report low-pitched wheezes, and vice versa. In fact, the difference between pure sinusoidal low-pitched wheezes and the more complex waveform of rhonchi is a subject of controversy and there is still some discrepancy in the literature [9, 10]. Therefore, and since bronchodilators act on airway muscles, but not on secretions, this study focuses only on CAS with a pitch above 200 Hz for assessing BDR [9, 11].

Conventional techniques to assess the presence of CAS, such as manual auscultation and respiratory questionnaires, are highly dependent on the subjectivity of the physicians and patients involved. In this sense, the automatic analysis of CAS provides objective, quantitative, and complementary information that may contribute to improving the assessment of asthma.

The association between CAS, airway obstruction severity, and BDR has been reported in several previous studies using conventional techniques [11–15]. On the other hand, the automatic detection and analysis of CAS has increased the objectivity of auscultation. Early studies used the power spectrum for analyzing CAS patterns [16, 17], quantifying the number of CAS [18, 19], or estimating the duration and frequency parameters of CAS [20–23]. More recently, time-frequency analysis has been used to calculate CAS features more precisely than with the power spectrum [24–28]. Some CAS features have been related to airway obstruction severity and BDR in patients with obstructive pulmonary diseases [19, 20, 22, 23, 29–31].

The lack of a standard method for CAS recording and analysis has led to several different CAS analysis approaches, with some methodological issues that could limit the potential of CAS analysis in clinical practice. Typically, respiratory sound signals have been recorded either at maintained flow levels or during forced expiratory maneuvers. However, CAS only appear above a critical flow, which depends on several factors and varies between people [32]. Therefore, as we previously described [33], CAS may be present over a wide range of flow levels. Furthermore, using only one microphone placed over the trachea, as in [17, 22, 29–31], could lead to a failure to detect changes in the structure of smaller airways after treatment.

Spectrogram, which has been the most widely used technique for CAS analysis, has poor resolution and could lead to an underestimation of CAS, as we demonstrated in a previous work [34]. Further, the intensity of CAS has rarely been analyzed, unlike their duration and frequency. Moreover, other CAS analysis methods have been applied to datasets in which background noises and artefacts had been manually rejected beforehand. Therefore, there is a lack of robust methodology in the analysis of CAS that can be directly applied to recorded respiratory sound signals. To date, an approach that considers all these aspects of CAS analysis has not been proposed.

In this study, we propose an integrated approach to CAS analysis for the assessment of BDR in asthma patients. This new approach includes a multichannel recording of respiratory sounds using four contact microphones and a progressive respiratory maneuver with variable flow that enables the analysis of CAS at several flow levels. This approach also includes our recently proposed method for CAS segmentation, based on the Hilbert spectrum [34], which allows CAS to be fully characterized with regard to duration, mean frequency, and intensity. Furthermore, we analyze CAS directly from the recorded signals, without the prior removal of other sounds and noises. Accordingly, we trained and validated a high performance classifier to retain only CAS and avoid the analysis of other sounds.

The proposed novel and robust approach to CAS analysis has been applied to asthma patients in order to assess BDR in terms of acoustic parameters. We suggest that this approach to CAS analysis provides distinct and complementary information about BDR, beyond that provided by spirometry, and could contribute to improving the stratification of BDR in asthma patients.

## Materials and methods

### Ethics statement

The study was conducted in the Respiratory Function Laboratory at Germans Trias i Pujol University Hospital and approved by the Human Research Ethics Committee of the hospital. All participants gave written informed consent, following the World Medical Association’s Declaration of Helsinki on Ethical Principles for Medical Research Involving Human Subjects.

### Study population

Asthma patients who came to the Pulmonology Service at Germans Trias i Pujol University Hospital, from March 2011 to May 2015, for regular examinations were recruited for performing the BDR test and participating in the respiratory sound recording and analysis study.

BDR was measured by spirometry and respiratory sounds were recorded, before and after bronchodilator administration, in 54 asthma patients. The patients were asked to withhold any bronchodilator medication overnight (at least twelve hours) prior to testing [1]. An increase in FEV_1_ of >12% and >200 mL from the baseline was considered to be a positive BDR (BDR+) [1], otherwise it was considered to be a negative BDR (BDR-). From the 54 recruited patients (17 BDR+ and 37 BDR-), 37 patients (15 BDR+ and 22 BDR-) who had baseline FEV_1_<80%—that is, a risk factor for asthma exacerbations [1]—were considered eligible for the CAS analysis study.

Since this study focuses on CAS analysis, and CAS are not present in all asthma patients [1], the respiratory sound signals of the 37 eligible patients were audiovisually inspected to distinguish between patients with and without CAS, at least at one of the recorded respiratory sound channels, and either at baseline or after bronchodilator administration. Due to the difficulty in determining the true pitch of CAS by audiovisual inspection of respiratory sound signals, no limit was considered for the pitch of CAS in this stage of the study. From the 37 eligible patients, 20 had presence of CAS (7 BDR+ and 13 BDR-) and were included in the CAS analysis study.

In addition, 5 control participants were recruited from healthy subjects who had never been diagnosed with any chronic respiratory disease and had normal baseline FEV_1_.

### Respiratory sound and flow recording protocol

**Fig 1:**
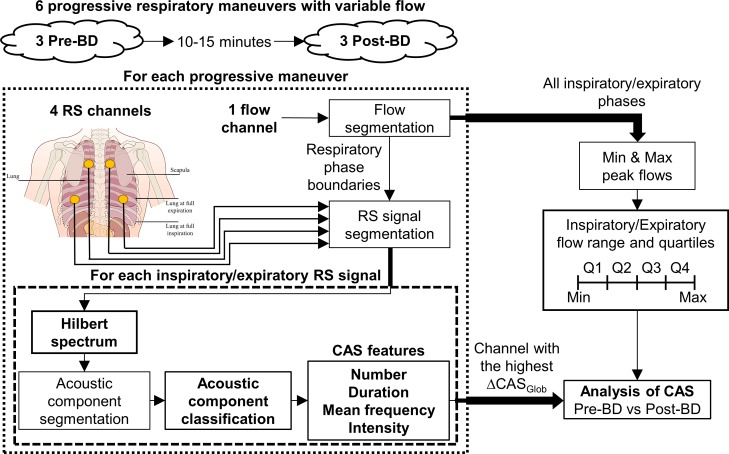
Flow chart of the proposed respiratory sound recording and CAS analysis approach. BD, bronchodilator; CAS, continuous adventitious sounds; Q1-Q4, flow quartiles; RS, respiratory sounds; ΔCAS_Glob_, change in the number of CAS after bronchodilation.

Four piezoelectric contact microphones were used for recording respiratory sounds (TSD108, Biopac^®^). Microphones were placed on the surface of the subjects’ backs, at the base and near the upper lobe of the two lungs (Fig 1). Respiratory flow was recorded simultaneously with respiratory sounds, using a pneumotachograph (TSD107B, Biopac^®^). All signals were sampled at 12500 samples/s (MP150, Biopac^®^) and, after digitalization, the respiratory sound signals were band-pass filtered between 70 Hz and 2000 Hz.

Respiratory sound signals from the back are affected by the low-pass filtering effect of the chest [35], and wheezes barely appear at frequencies above 1600 Hz [36]. Accordingly, respiratory sound signals were decimated by four to a sampling rate of 3125 Hz.

The subjects were placed in a sitting position and performed six progressive maneuvers with variable flow, three pre-bronchodilator and three post-bronchodilator. They were guided by a video that showed a sequence of arrows spreading out, either upwards (inspiration) and downwards (expiration). The duration and length of the arrows determined the rate and depth of breathing and these varied over the course of each maneuver, thus encouraging subjects to start breathing normally, becoming progressively faster and deeper until they reached the deepest breaths they were able to take, and finally returning to normal breathing. Around twenty respiratory cycles were obtained from each progressive maneuver.

### Respiratory sound and flow signal processing

#### Segmentation of respiratory phases and definition of flow quartiles

**Fig 2:**
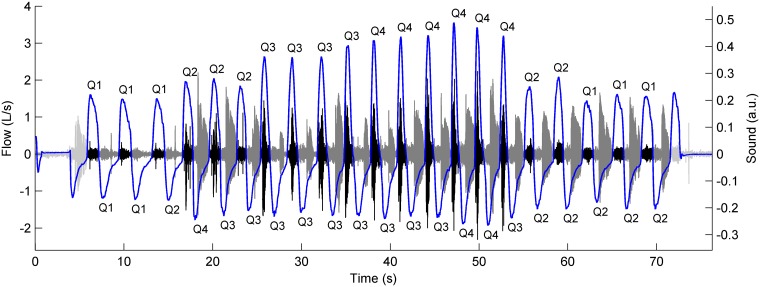
Respiratory sounds and flows recorded during the performance of a progressive maneuver at baseline. Segmented respiratory phases are highlighted in black (inspiration) and dark grey (expiration). Q1-Q4 labels indicate the flow quartile assigned to each respiratory phase.

The respiratory phases were segmented by means of a zero-crossing detector applied to the flow signals, as described in our previous work [37]. After segmenting the respiratory phases from a subject’s three pre-bronchodilator and three post-bronchodilator flow signals, the inspiratory and expiratory flow ranges were calculated as the differences between the maximum and minimum peak flows of all the inspiratory and expiratory phases, respectively. Flow ranges were divided into four quartiles, Q1-Q4, with Q1 including the lowest flows. In this way, a flow quartile was assigned to each respiratory phase, according to its peak flow (Fig 2).

#### Acoustic component segmentation and characterization

After having segmented all the respiratory phases, the corresponding respiratory sound signals from the four recorded channels of a subject were analyzed, for acoustic component segmentation, using a new method that we have recently described in two previous studies, based on the analysis of the instantaneous frequency (IF) and envelope (IE) sequences [33], and the Hilbert spectrum time-frequency distribution [34]. The method is briefly described in this section.

First, our automatic CAS detection algorithm [33] was applied to each respiratory sound signal. Based on the fact that the IF dispersion markedly decreases when CAS appear within a respiratory sound signal, that algorithm detected respiratory sound signal segments that were more likely to contain CAS. To do that, each respiratory sound signal was decomposed by ensemble empirical mode decomposition into a set of narrowband components for which the IF and IE sequences were calculated by means of the Hilbert transform. Then, a number of dispersion-based criteria were used on the IF sequences to detect respiratory sound signal segments with a lower IF dispersion. Each detected segment was characterized by means of a set of features extracted from the IF and IE sequences, including the mean and standard deviation IF. These features were used to classify respiratory sound signal segments as CAS segments or normal segments, using a support vector machine classifier.

**Fig 3:**
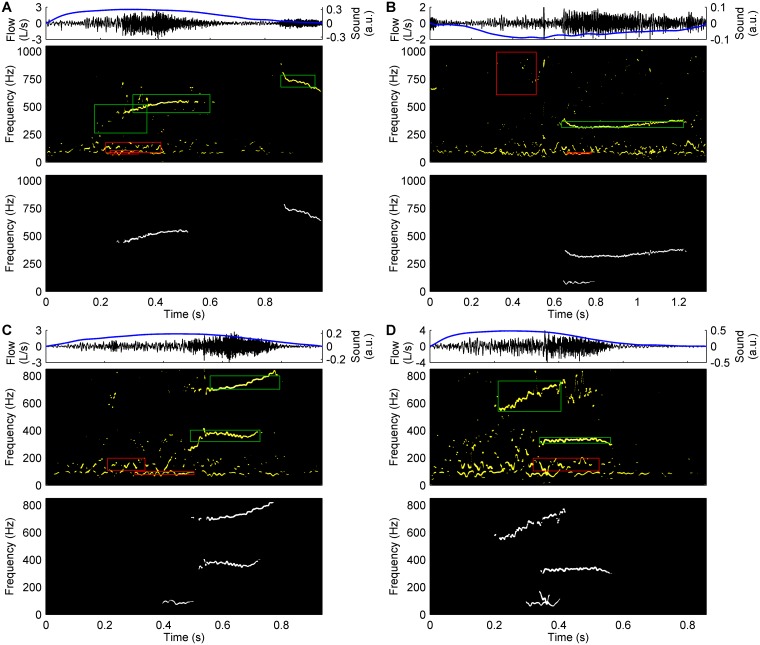
Segmentation of acoustic components from the Hilbert spectrum. For each subfigure: flow and respiratory sound signals of a respiratory cycle (top), the Hilbert spectrum of the respiratory sound signal (middle), and the acoustic components segmented using our acoustic component segmentation method [34] (bottom). In the Hilbert spectrums, CAS areas are plotted in green, and normal areas are plotted in red. A) Two inspiratory CAS components occurring at different times (multiple monophonic CAS). B) A single expiratory CAS component. C) D) Inspiratory CAS components overlapping in time (polyphonic CAS). Subfigures B, C, and D also show some low-pitched non-CAS components that correspond to normal respiratory sounds but are similar to CAS components, and therefore are segmented by our acoustic component segmentation method.

After detecting CAS and normal segments within a respiratory sound signal, the acoustic component segmentation was performed [34]. First, the IF and IE sequences were rearranged into an array to obtain the Hilbert spectrum time-frequency distribution. Then, the previously calculated mean and standard deviation IF of each detected segment were used to mark out an analysis area in the Hilbert spectrum. Each analysis area was centred on the mean IF, had a frequency width of twice the standard deviation IF, and had the same duration as its corresponding segment. Analysis areas corresponding to CAS segments (CAS areas) were more likely to contain acoustic components corresponding to CAS (CAS components), whereas analysis areas corresponding to normal segments (normal areas) were more likely to contain acoustic components corresponding to other sounds (non-CAS components) (Fig 3). In any case, since CAS appeared in the Hilbert spectrum as thin ridges where CAS energy concentrated (Fig 3), any acoustic components represented as ridges were automatically segmented using a region-growing algorithm and region-linking criteria. Both normal and CAS areas were considered for seed point searching and local region growing, but different region-linking criteria were used, depending on the type of analysis area, to guarantee the temporal and frequency continuity of the segmented acoustic components.

In respiratory cycles containing multiple CAS components, either occurring at different times (multiple monophonic CAS) or overlapping in time (polyphonic CAS), each CAS component appeared in a different analysis area and was segmented separately (Fig 3A, 3C and 3D). Therefore, multiple monophonic CAS or polyphonic CAS counted as two (or more) CAS components.

Our algorithm calculated a number of parameters for each segmented acoustic component. Duration (*D*) was calculated as the difference between the highest and the lowest time index among all the points belonging to the segmented acoustic component. Intensity (*I*) was calculated as in Eq 1.

$$I\left(dB\right)=10log_{10}\frac{\sum_{\left(t,f\right)\in ridge}H{\left(t,f\right)}^{2}}{\sum_{\left(t,f\right)\notin ridge}H{\left(t,f\right)}^{2}}$$ (1)

where *H*(*t*,*f*) is the Hilbert spectrum, *t* is the time index, *f* is the frequency index, (*t*,*f*)∈*ridge* are points of *H*(*t*,*f*) that are inside the segmented acoustic component, and (*t*,*f*)∉*ridge* are points of *H*(*t*,*f*) that are outside the segmented acoustic component.

Some frequency parameters, including the mean frequency (*F*_*Mean*_) and the standard deviation frequency (*σ*_*F*_), were calculated as in Eqs 2 and 3.

$$F_{Mean}\left(Hz\right)=\frac{\sum_{\left(t,f\right)\in ridge}fH\left(t,f\right)}{\sum_{\left(t,f\right)\in ridge}H\left(t,f\right)}$$ (2)

$$\sigma_{F}\left(Hz\right)=\sqrt{\frac{\sum_{\left(t,f\right)\in ridge}{\left(f-F_{mean}\right)}^{2}H\left(t,f\right)}{\sum_{\left(t,f\right)\in ridge}H\left(t,f\right)}}$$ (3)

Moreover, the mean point-by-point *σ*_*F*_ ($\bar{\sigma_{F}}$) was calculated as the average value of the standard deviation frequency at each time index along the segmented acoustic component.

According to the definition of CAS [8], only acoustic components with *D*>100 ms were retained, regardless of their *F*_*Mean*_, as described in [34].

### Classification of acoustic components

**Fig 4:**
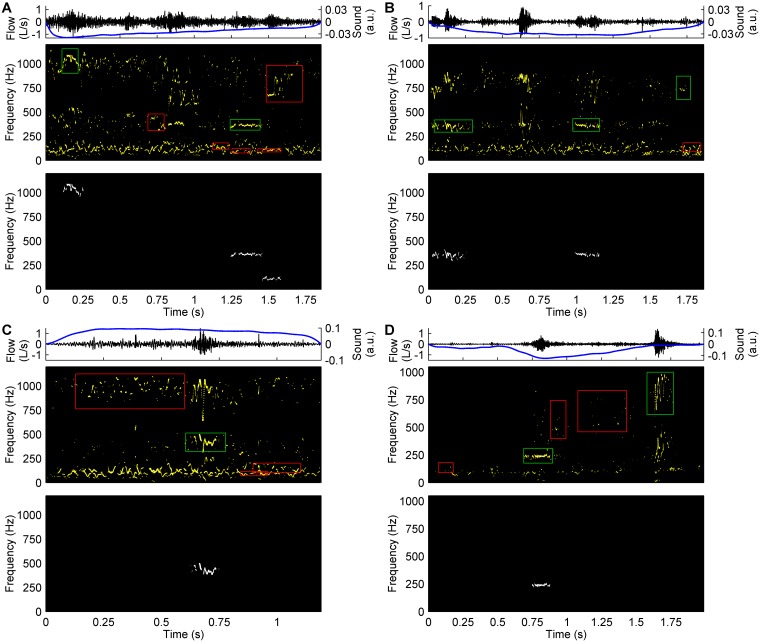
Background noises from the clinical environment. Four examples of respiratory cycles containing background noises, including talking (A), footsteps (B), banging (C), and furniture dragging (D), from the clinical environment. These sounds appear in the Hilbert spectrum as high-pitched (above 200 Hz) acoustic components with a certain degree of temporal and frequency continuity, similar to CAS components.

Besides CAS components, non-CAS components corresponding to other sounds, such as normal respiratory sounds (Fig 3B, 3C and 3D) or background noises from the clinical environment (Fig 4), often appear in the Hilbert spectrum as ridges similar to those described by CAS. These non-CAS components could be segmented by our acoustic component segmentation method, thus resulting in an overestimation of the presence of CAS components.

In this study, a support vector machine classifier [38, 39] has been trained and validated in order to distinguish CAS components from non-CAS components. This classifier is the last stage of our proposed integrated approach to CAS analysis, and complements our acoustic component segmentation method [33, 34].

In order to form the training and testing datasets for the classifier, our acoustic component segmentation method was applied to the respiratory sound signals from the four recorded channels of the twenty-five subjects. For each subject, the channel with the highest number of segmented acoustic components, during the pre-bronchodilator and post-bronchodilator progressive maneuvers, was selected.

**Fig 5:**
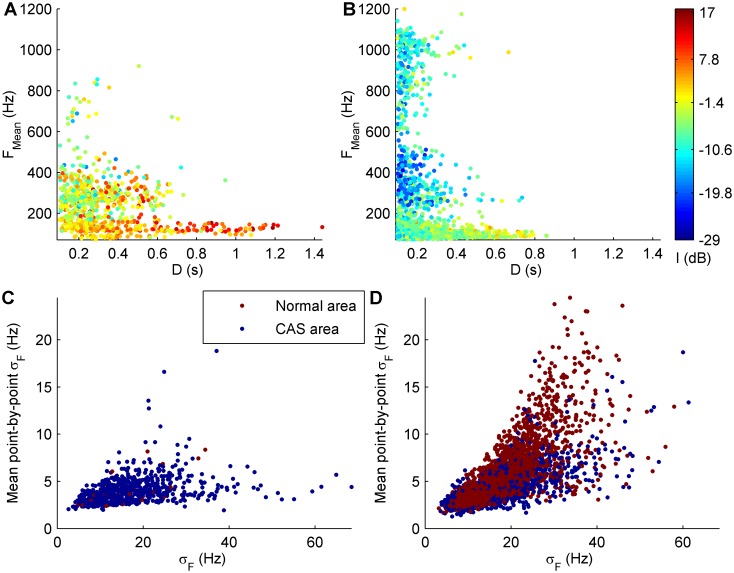
Features of the classification dataset. In all subfigures, each circle represents an acoustic component of the classification dataset. Subfigures A and B show the duration (*D*), mean frequency (*F*_*Mean*_), and intensity (*I*) of CAS and non-CAS components, respectively. Subfigures C and D show the standard deviation frequency (*σ*_*F*_), mean point-by-point *σ*_*F*_ ($\bar{\sigma_{F}}$), and type of analysis area of CAS and non-CAS components, respectively.

A total of 5149 acoustic components were obtained from the selected channels of the study subjects and formed the classification dataset. These acoustic components were labeled as CAS components (816 components) or non-CAS components (4333 components) by audiovisual inspection of the corresponding respiratory sound signals. Input feature vectors included the essential features of CAS (*D*, *F*_*Mean*_, and *I*), *σ*_*F*_, $\bar{\sigma_{F}}$, and the type of analysis area (Fig 5). These parameters were calculated by our acoustic component segmentation method [34] and have been described in the previous section.

CAS components had lower *σ*_*F*_ and $\bar{\sigma_{F}}$ than non-CAS components (Fig 5C and 5D). These two parameters represent the degree of energy dispersion of the acoustic components in the Hilbert spectrum. As described in the previous section, CAS have a sinusoidal-like waveform, and therefore their energy is more concentrated (lower *σ*_*F*_ and lower $\bar{\sigma_{F}}$) around the *F*_*Mean*_ than the energy of non-CAS components, which is more scattered (higher *σ*_*F*_ and higher $\bar{\sigma_{F}}$). Moreover, CAS components were more likely to be segmented from CAS areas (Fig 5C) than non-CAS components, most of which were segmented from normal areas (Fig 5D).

The imbalance between the number of CAS components (16% of the classification dataset) and non-CAS components (84% of the classification dataset) represents the normal situation in respiratory sound analysis. The sources of this imbalance were normal respiratory sounds and background noises. Different from CAS, which do not appear in all respiratory cycles, normal respiratory sounds are always present in respiratory sound signals. These sounds have a frequency band from around 70 Hz up to 200–250 Hz [8, 35]. Since our acoustic component segmentation method did not set any limit for the *F*_*Mean*_ of the acoustic components, 3670 non-CAS components (71% of the classification dataset) with *F*_*Mean*_<200 Hz (Fig 5B) were mainly segmented from normal respiratory sounds, as those shown in Figs 3B–3D and 4A. These non-CAS components had lower *I* than low-pitched CAS components (Fig 5A). On the other hand, respiratory sound signals were recorded in a room next door to the lung function laboratory and the clinic’s waiting room at the Germans Trias i Pujol Hospital. Even though the room was quiet, many noises from outside the room, mainly including those shown in Fig 4, were easily listened to and often appeared in the recorded respiratory sound signals. This is evidenced in Fig 5B, which shows 663 non-CAS components (13% of the classification dataset) corresponding to background noises with low *D* (mostly below 0.3 s), high *F*_*Mean*_ (above 200 Hz), and much lower *I* than CAS components.

The dataset was randomly partitioned into training (65%, 3349 components) and testing (35%, 1800 components) subsets, each one containing a proportionate share of the CAS and non-CAS components from each subject. The training subset was used to find the optimum parameters of the classifier. In binary classification, the goal of a support vector machine classifier is to find the optimal hyperplane that separates all data points into two classes. However, a simple hyperplane cannot be found in the original feature space when the relation between classification labels and input features is nonlinear. In that case, a nonlinear kernel function is used to map input features into a higher dimensional space, where linear classification can be performed. As a rule of thumb [39], if the number of input features is large there is no need to map data to a higher dimensional space. On the contrary, if the number of features is small and much lower than the number of instances in the classification dataset, using a nonlinear kernel improves the performance of the classifier. Since, in this study, feature vectors had 6 components and the training subset had 3349 acoustic components, a nonlinear radial basis function kernel was used to train the classifier. The radial basis function is the most commonly used kernel among nonlinear kernels, since it has high performance in many classification problems and has less parameters than the polynomial kernel, which facilitates the identification of the optimum parameters [38].

Using a radial basis function kernel required identifying two parameters: the penalty parameter, *C*, and the kernel scale, *σ*. The standard grid-search method with cross-validation was used to identify the optimum parameters [38]. Several combinations of exponentially growing sequences of *C* (e^-2^, e^-1.75^, e^-1.5^, …, e^6^) and *σ* (e^-0.5^, e^-0.25^, e^0^, …, e^3.5^) were used as initial points for finding local minimums of the 10-fold cross-validation loss function. Each (*C*, *σ*) pair which produced a local minimum was used to train a support vector machine classifier from the training subset. The resulting classifiers were validated using the testing subset and their performance was evaluated in terms of accuracy, sensitivity, specificity, and positive predictive value.

The training and validation steps for all the (*C*, *σ*) pairs were repeated for one hundred different random dataset partitions, so that the results were independent of the initial partition. For each (*C*, *σ*) pair, the mean and standard deviation of the performance parameters along all partitions were calculated. Since the number of CAS components in the dataset was much lower than the number of non-CAS components, sensitivity and positive predictive value in detecting CAS components were the parameters used for selecting the optimum (*C*, *σ*) pair. Accordingly, the (*C*, *σ*) pair that achieved the highest summation of the mean testing sensitivity and positive predictive value was selected. Finally, the support vector machine classifier that was trained with the selected (*C*, *σ*) pair, and reached the highest summation of the testing sensitivity and positive predictive value, among all partitions, was chosen to distinguish CAS components from non-CAS components.

### Channel selection and CAS component analysis

The acoustic component classifier was applied to all acoustic components segmented from the four recorded channels of the twenty-five subjects. As mentioned in the introduction, most low-pitched CAS are related to secretions and are not affected by bronchodilators, so they should not be considered for BDR assessment. Accordingly, in this study, all CAS components with *F*_*Mean*_<200 Hz were automatically rejected. This cut-off has been widely described in the literature [11, 35, 40, 41] and has been recently recommended by the task force for lung sounds from the European Respiratory Society [9, 10]. Nevertheless, the frequency cut-off for distinguishing low-pitched CAS from high-pitched CAS is still a source of debate.

For each recorded channel of a subject, the number of inspiratory or expiratory CAS, either pre-bronchodilator or post-bronchodilator, was expressed as in Eq 4.

$$\#CAS_{X_{i}}\left(\%\right)=100*\frac{\#CAS\;components_{X_{i}}}{\#Respiratory\;cycles_{X_{i}}}\;i=1,2,3,4\;\;X=\left\{InsPre-BD,InsPost-BD,ExpPre-BD,ExpPost-BD\right\}$$ (4)

where the # symbol denotes “number of”. Respiratory cycles containing multiple monophonic CAS or polyphonic CAS contributed more than one CAS component to the number of CAS. Moreover, the change in the number of CAS after bronchodilator administration was calculated as in Eqs 5 and 6.

$$\Delta CAS_{Ins}\left(\%\right)=\left|\#CAS_{InsPost-BD}-\#CAS_{InsPre-BD}\right|$$

$$\Delta CAS_{Exp}\left(\%\right)=\left|\#CAS_{ExpPost-BD}-\#CAS_{ExpPre-BD}\right|$$

$$\Delta CAS_{Glob}\left(\%\right)=\left|\#CAS_{GlobPost-BD}-\#CAS_{GlobPre-BD}\right|=\text{max}\left\{\Delta CAS_{Ins},\Delta CAS_{Exp}\right\}$$ (5)

$$n\Delta CAS_{Glob}\left(\%\right)=100*\frac{\Delta CAS_{Glob}}{\#CAS_{GlobPre-BD}}$$ (6)

*ΔCAS*_*Glob*_ and *nΔCAS*_*Glob*_ are two ways of expressing the change in the number of CAS. *ΔCAS*_*Glob*_ is simply the absolute value of the difference between the number of CAS post-bronchodilator and the number of CAS pre-bronchodilator, whereas in *nΔCAS*_*Glob*_ that difference is normalized and expressed as a percentage of the number of CAS pre-bronchodilator. For each subject, the channel with the highest *ΔCAS*_*Glob*_ was selected to assess BDR in terms of number of CAS. Besides the number of CAS, we also analyzed the *F*_*Mean*_, *D*, and *I* of CAS components, since these parameters could be different between the two groups of asthma patients and could vary after bronchodilator administration.

### Statistical analysis

Statistical analyses were performed using the Statistics and Machine Learning Toolbox^™^ in Matlab^®^ R2014a.

Differences in the anthropometric and spirometry variables of the three groups of subjects (controls, BDR- patients, and BDR+ patients) were analyzed using non-parametric Kruskal-Wallis tests. For those variables showing a significant difference (p<0.05), multiple post hoc group comparisons were performed, using the Bonferroni adjustment, to determine where the difference between the three groups was.

The change in the number of CAS of the study subjects was analyzed in two ways. First, differences between *#CAS*_*GlobPre-BD*_ and *#CAS*_*GlobPost-BD*_ were analyzed separately for each group of subjects, using nonparametric Wilcoxon rank sum tests, since the number of subjects within each group was not large enough to test normality and use a parametric test. Second, a Wilcoxon rank sum test was also used to analyze the difference in *nΔCAS*_*Glob*_ between BDR+ patients and BDR- patients. More details about statistical analyses of the number of CAS and CAS features are provided later in the text.

## Results and discussion

### Study subjects

Anthropometric and spirometry data of the twenty-five study subjects are shown in Table 1.

**Table 1:** Anthropometric and spirometry data of the study subjects.

Variables	Controls	BDR- patients	BDR+ patients	p-value
**Anthropometric data**				
Total number	5	13	7	
Gender (male:female)	3:2	5:8	3:4	
Age (years)	31 (26–50)	53 (37–64)	42 (34–66)	0.359
Height (m)	1.74 (1.70–1.82)^†^	1.67 (1.62–1.71)	1.62 (1.54–1.66)^†^	0.03
BMI (kg/m^2^)	23.5 (20.48–26.66)	25.15 (21.73–29.43)	22.22 (20.25–25.6)	0.463
**Spirometry data**				
FEV_1_ (% of predicted)	97 (90–99)*^†^	72 (64–75)*	51 (42–60)^†^	0.00018
ΔFEV_1_ (% from baseline)	1 (0–2)^†^	8 (6–12)^§^	41 (26–51)^†^^§^	<0.0001
ΔFEV_1_ (% of predicted)	1 (0–2)^†^	6 (4–8)^§^	19 (15–25)^†^^§^	<0.0001
FVC (% of predicted)	97 (92–104)^†^	73 (68–88)	62 (61–77)^†^	0.008
ΔFVC (% from baseline)	-3 (-4–-1.5)^†^	3 (2–9)^§^	21 (14–36)^†^^§^	0.00015
FEV_1_/FVC pre-BD (%)	80 (75–84)^†^	69 (63–76)	55 (52–63)^†^	0.002
FEV_1_/FVC post-BD (%)	83 (78–86)^†^	72 (65–81)	68 (60–72)^†^	0.031

p-values were calculated using non-parametric Kruskal-Wallis tests. Multiple post hoc comparison tests were performed using the Bonferroni adjustment.

* Difference between controls and BDR- patients (p<0.05)

^†^ Difference between controls and BDR+ patients (p<0.05)

^§^ Difference between BDR- and BDR+ patients (p<0.05)

BMI, body mass index; FEV_1_, forced expiratory volume in 1 second; FVC, forced vital capacity; BD, bronchodilator; BDR-/+, negative/positive bronchodilator response. All values are presented as median (interquartile range).

The subjects did not differ significantly in age or body mass index. There was a significant difference between the height of controls and the height of BDR+ patients. Although lung function is affected by height, which is a determinant of lung size and airway dimension [42, 43], expressing FEV_1_ and FVC as percentages of predicted values eliminates the influence of height. Predicted values are calculated using reference equations that are a function of sex, age, and height and are derived from large healthy populations [2]. However, the standard BDR criterion, based on the percentage change in FEV_1_ from baseline, is dependent on the baseline FEV_1_, and therefore on height. For this reason, the use of a BDR criterion based on the percentage of predicted FEV_1_ was proposed by the European Respiratory Society [44], and supported in some previous studies [42, 43, 45]. When expressed as a percentage of predicted FEV_1_, a change that is >9% is considered to be a positive BDR. In this study, changes in FEV_1_ of the three groups of subjects did not differ for the two BDR criteria, as shown in Table 1.

As expected, there were significant differences in all the spirometry parameters between controls and BDR+ patients, with the latter having lower baseline FEV_1_, FVC, and FEV_1_/FVC values and higher changes in all these parameters after bronchodilation. Regarding BDR- patients, there were no significant differences in baseline FVC and baseline FEV_1_/FVC between them and either controls or BDR+ patients. Furthermore, although baseline FEV_1_ was significantly lower in BDR- patients than in controls, there were no significant differences in the change in FEV_1_ between the two groups. However, the change in FEV_1_ was significantly lower in BDR- patients than in BDR+ patients. These results show that BDR- patients are of special clinical interest since, although their baseline spirometry parameters may range from those of controls to those of BDR+ patients, their lung function does not significantly change after bronchodilation. In this group, the analysis of CAS before and after bronchodilator administration could provide useful complementary information about BDR, from the point of view of pulmonary acoustics.

### Classification of acoustic components

The (*C*, *σ*) pair with the highest summation of the mean testing sensitivity (85.9% ± 1.6%) and positive predictive value (88.9% ± 1.8%) for all dataset partitions was the (1.92·10^4^, 12.66) pair. Among the support vector machine classifiers trained with these optimum parameters and the different dataset partitions, we selected the classifier that achieved the highest summation of the testing sensitivity (87.7%) and positive predictive value (92.2%). As shown in Table 2, the selected support vector machine classifier achieved high performance parameters for differentiating CAS components from non-CAS components.

**Table 2:** Performance of the acoustic component classifier.

	All components		Components with *F*_*Mean*_>200 Hz
	Testing	Overall	Overall
True positive	249	719	370
True negative	1495	4260	620
False positive	21	73	43
False negative	35	97	65
Sensitivity (%)	87.7	88.1	85.1
Specificity (%)	98.6	98.3	93.5
Positive predictive value (%)	92.2	90.8	89.6
Accuracy (%)	96.9	96.7	90.2

True positive/negative, CAS/non-CAS components properly classified; False positive/negative, non-CAS/CAS components misclassified; *F*_*Mean*_, Mean frequency.

Table 2 also shows the performance of the acoustic component classifier only for components with *F*_*Mean*_>200 Hz, which are the acoustic components considered for BDR assessment in this study. Although in this case the performance of the classifier was slightly lower than that for all acoustic components, it was still high. It is important to note that the classification dataset of this study included respiratory sound signals in which background noises and artefacts were not rejected beforehand, that is just like they were recorded in a clinical environment. Moreover, as described in [34], our acoustic component segmentation method is less dependent on amplitude criteria than most previous algorithms for CAS detection. Therefore, our method allowed us to detect CAS components with widely different intensities, including many weak CAS components, whereas previous algorithms for CAS detection focused on CAS components with higher intensity. In this sense, the 435 CAS components with *F*_*Mean*_>200 Hz included in our classification dataset had intensities between -18.1 and 16.9 dB (-2 dB ± 5.7 dB). From these CAS components, the 65 false negatives corresponded to weak CAS components, with a very low intensity of -10 dB ± 3.6 dB. Even so, our classifier managed to detect not only loud CAS components, but also many CAS components with low intensity.

### Analysis of the number of CAS to assess BDR

After segmenting and classifying the acoustic components from the four recorded channels of the study subjects, the channel with the highest *ΔCAS*_*Glob*_ was selected for each subject. The channel selection for the 20 asthma patients was the following: lower left in 7 patients, lower right in 3 patients, upper left in 7 patients, and upper right in 3 patients. Moreover, 15 of the 20 asthma patients had the highest *ΔCAS*_*Glob*_ in the inspiratory phase, whereas 5 asthma patients had the highest *ΔCAS*_*Glob*_ in the expiratory phase. These results reflect the diffusion and non-uniform distribution of airway obstruction in asthma. Since CAS are associated with airway obstruction, these may appear at any point throughout the lungs, from central to peripheral airways, and during inspiration, expiration, or both [46].

A key point of our proposed approach to CAS analysis is the progressive respiratory maneuver with variable flow. This is a real advantage as compared to previous studies, in which CAS were analyzed at constant flows or during forced expiratory maneuvers. It has been reported that forced expiratory wheezes have low specificity for the clinical diagnosis of asthma, since many wheezes may also appear in healthy people during forced expiratory maneuvers [12, 18]. On the other hand, a flow-dependent analysis of CAS is important because CAS only appear above a critical flow, and this depends on the mechanical properties of airways, which in turn vary between people [32]. In this sense, the progressive respiratory maneuver, which is easier to perform than the forced expiratory maneuver, allows CAS to be analyzed at a wide range of flow levels, from normal to forced breathing.

**Fig 6:**
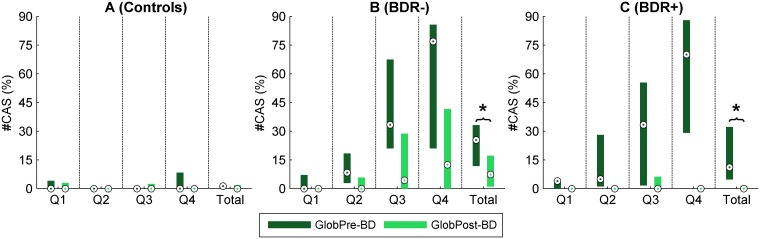
Number of CAS of the three groups of subjects. Box plot distributions of the number of CAS at baseline (*#CAS*_*GlobPre-BD*_) and after bronchodilator administration (*#CAS*_*GlobPost-BD*_) for the flow quartiles (Q1-Q4) and the total flow range of controls (A), BDR- patients (B), and BDR+ patients (C). Circles within boxes = medians, boxes = interquartile ranges. Comparisons between *#CAS*_*GlobPre-BD*_ and *#CAS*_*GlobPost-BD*_ in the total flow range of BDR- and BDR+ groups were made using Wilcoxon rank sum tests. Significant differences (p<0.05) are indicated with an asterisk.

Fig 6 shows *#CAS*_*GlobPre-BD*_ and *#CAS*_*GlobPost-BD*_ for the flow quartiles and the total flow range of the three groups of subjects.

As expected, the number of CAS increased with flow in both BDR- and BDR+ patients, indicating that the greater the flow the higher the possibility of CAS appearing (Fig 6B and 6C). Even so, CAS appeared over the wide range of flow levels.

In controls, *#CAS*_*GlobPre-BD*_ and *#CAS*_*GlobPost-BD*_ were nearly zero, which agreed with the absence of airway obstruction in these subjects. However, in both BDR- and BDR+ patients, *#CAS*_*GlobPre-BD*_ was appreciable, suggesting some degree of airway obstruction. In BDR+ patients, *#CAS*_*GlobPost-BD*_ was not only significantly lower than *#CAS*_*GlobPre-BD*_, but nearly zero, which means that CAS almost completely disappeared (Fig 6C). Therefore, BDR+ patients also had a high BDR in terms of number of CAS. Nevertheless, in BDR- patients, *#CAS*_*GlobPost-BD*_ was significantly lower than *#CAS*_*GlobPre-BD*_ (Fig 6B) but still appreciable, indicating some degree of airway obstruction reversibility, but lower than that of BDR+ patients.

**Fig 7:**
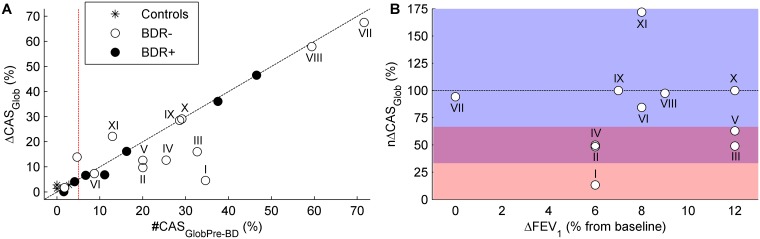
Individual change in the number of CAS after bronchodilator administration. A) Number of CAS at baseline (*#CAS*_*GlobPre-BD*_) and change in the number of CAS (*ΔCAS*_*Glob*_) of each study subject. The dashed line is the line of equality (*ΔCAS*_*Glob*_ = *#CAS*_*GlobPre-BD*_). That is, points on the line corresponded to subjects whose CAS completely disappeared after bronchodilation. Points under the line corresponded to subjects whose number of CAS was partially reduced after bronchodilation. Points above the line corresponded to subjects whose number of CAS increased after bronchodilation. B) *ΔFEV*_*1*_ and *nΔCAS*_*Glob*_ of BDR- patients whose *#CAS*_*GlobPre-BD*_ was >5% (white points to the right of the red dashed line in A). Points in A and B corresponding to the same subject have been labelled with the same Roman numeral. As in A, the dashed line in B represents *ΔCAS*_*Glob*_ = *#CAS*_*GlobPre-BD*_, which means *nΔCAS*_*Glob*_ = 100% (see Eq 6). Pink area: 0%<*nΔCAS*_*Glob*_<33%, purple area: 33%<*nΔCAS*_*Glob*_<66%, blue area: 66%<*nΔCAS*_*Glob*_.

Due to the high degree of inter-subject variability in *#CAS*_*GlobPre-BD*_, and therefore in *ΔCAS*_*Glob*_, these parameters were analyzed separately for each subject (Fig 7).

Only subjects with *#CAS*_*GlobPre-BD*_>5% were considered for CAS analysis, as indicated in Fig 7A (red dashed line). Two aspects were taken into consideration to fix this threshold. First, around 10% of detected CAS were false positives (see positive predictive value in Table 2). Further, as shown in Fig 6B and 6C, *#CAS*_*GlobPre-BD*_ in the total flow range was mostly below 50% in asthma patients. Therefore, the proportion of *#CAS*_*GlobPre-BD*_ that corresponded to false CAS was around 5%. False CAS were not affected by bronchodilators, and therefore should not be considered for BDR assessment. Second, Fig 7A shows that all controls, who were healthy subjects and were not expected to have CAS, had *#CAS*_*GlobPre-BD*_<3%. Assuming that value as the normal *#CAS*_*GlobPre-BD*_ in healthy people, we considered 5% to be an appropriate lower limit for *#CAS*_*GlobPre-BD*_, so that it was indicative of some degree of airway obstruction. Moreover, this threshold prevented *nΔCAS*_*Glob*_ from taking meaningless values due to very low *#CAS*_*GlobPre-BD*_ values. Taking into consideration this criterion, all controls, 2 BDR- patients, and 2 BDR+ patients were not included in the CAS analysis (Fig 7A). Therefore, a total of 11 BDR- patients and 5 BDR+ patients were considered for the CAS analysis.

There was high variability in *#CAS*_*GlobPre-BD*_ and *ΔCAS*_*Glob*_ among asthma patients (Fig 7A). Even so, all BDR+ patients were consistently close to the line of equality (*ΔCAS*_*Glob*_ = *#CAS*_*GlobPre-BD*_) which, according to Eq 5, means that *#CAS*_*GlobPost-BD*_ was nearly zero in all these patients. However, BDR- patients exhibited varied responses in terms of number of CAS. The variability in responses from BDR- patients is better shown in Fig 7B. In this case, *nΔCAS*_*Glob*_ is used to counterbalance the high intersubject variability of *#CAS*_*GlobPre-BD*_. Although all these patients had a non-significant BDR in FEV_1_ (ΔFEV_1_≤12%), they showed a wide range of responses in the number of CAS, as expressed by *nΔCAS*_*Glob*_. Instead of fixing a cut-off for *nΔCAS*_*Glob*_, we propose the following categorization: low response (L1, 0%≤*nΔCAS*_*Glob*_≤33%), medium response (L2, 33%<*nΔCAS*_*Glob*_≤66%), and high response (L3, 66%<*nΔCAS*_*Glob*_). According to this categorization, 6 BDR- patients had high response, 4 BDR- patients had medium response, and 1 BDR- patient had low response in *nΔCAS*_*Glob*_.

**Fig 8:**
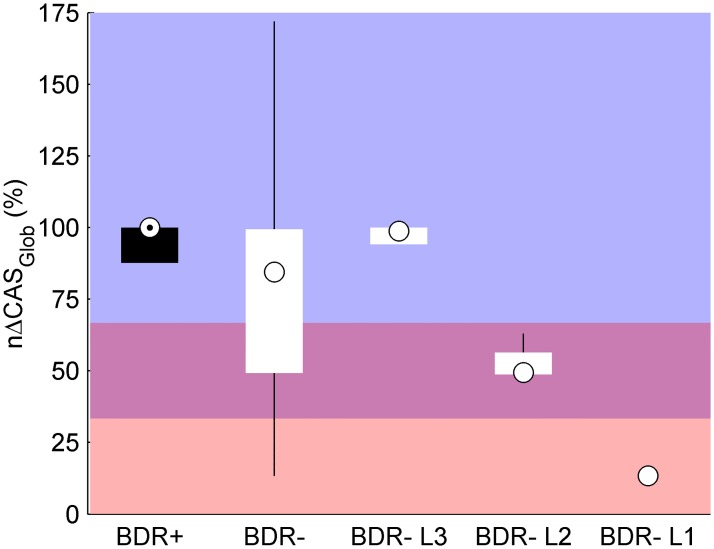
Population change in the number of CAS after bronchodilator administration. Boxplot distribution of the population change in the number of CAS. Circles within boxes = medians, boxes = interquartile ranges, whiskers = 1.5*interquartile ranges. Pink area = low response (L1), purple area = medium response (L2), blue area = high response (L3).

The population *nΔCAS*_*Glob*_ is shown in Fig 8, for both BDR+ and BDR- patients, as well as for the three subgroups of BDR- patients.

The difference in *nΔCAS*_*Glob*_ between BDR+ patients and BDR- patients was analyzed using the Wilcoxon rank sum test, since the number of subjects within each group (5 BDR+ and 11 BDR- patients) was not large enough to use a parametric test. Due to the high variability of the BDR- group, the difference in *nΔCAS*_*Glob*_ between the two groups was not significant (p = 0.2614). Differences in *nΔCAS*_*Glob*_ between BDR+ patients and the three subgroups of BDR- patients were analyzed using a Kruskal-Wallis test (p = 0.0223). Although the p-value indicated a significant difference between the four groups, subsequent post hoc comparisons did not detect significant differences between pairs of groups, presumably due to the small number of subjects per group (5 BDR+, 6 BDR- L3, 4 BDR- L2, and 1 BDR- L1). In any case, two aspects should be noted. First, BDR+ patients had a homogenous high response in *nΔCAS*_*Glob*_, which agreed with the positive BDR in FEV_1_, indicating high reversibility of airway obstruction. Second, the proposed categorization for *nΔCAS*_*Glob*_ allowed us to stratify BDR- patients into smaller and more consistent groups of response in terms of number of CAS. From the 11 BDR- patients, 6 (55%) had a high response in *nΔCAS*_*Glob*_, similar to BDR+ patients, whereas another 4 (36%) had a noteworthy medium response in *nΔCAS*_*Glob*_.

It is true that the prevalence of significant (high or medium) responses in *nΔCAS*_*Glob*_ might be favored by the fact that only patients who had CAS were included in this study, and bronchodilators are expected to reduce the number of CAS. However, CAS are not present in all asthma patients. In fact, only 11 of the 22 BDR- patients who had FEV_1_<80% (see Study population in the Materials and Methods section) had *#CAS*_*GlobPre-BD*_>5% and were considered for CAS analysis. Despite that, the 10 BDR- patients who had a medium or a high response in *nΔCAS*_*Glob*_ still represent a noteworthy share (45%) of the initial 22 BDR- patients.

The aforementioned results indicate that the proposed approach to CAS analysis is a very sensitive tool and provides useful information on BDR that is not provided by spirometry. Bronchodilators cause the airway muscles to relax, thus dilating and opening airways. The resulting bronchodilation reduces airway obstruction and helps to relieve difficult breathing and symptoms, such as CAS. These bronchodilator effects were evident in BDR+ patients, as measured not only by spirometry, which is the standard methodology for assessing BDR, but also by CAS analysis. However, BDR- patients did not show a significant BDR in terms of spirometry parameters. It is noteworthy that spirometry yields global measurements of pulmonary ventilation and may not be affected by local airway obstruction. In this regard, our proposed configuration for recording respiratory sounds using four contact microphones (posterior base and posterior upper lobe of the lungs) is a key point of the proposed approach to CAS analysis, since it provides a broader perspective of airway obstruction and allows us to detect local changes due to bronchodilator administration. In fact, we found an appreciable response, in terms of number of CAS, in most BDR- patients. The proposed multichannel configuration meets the CORSA guidelines [47] and includes the minimum number of sensors required for analyzing the distribution of CAS on the chest surface both laterally and vertically, without requiring a large number of sensors and the resulting larger amount of data. Indeed, in the literature, most studies have focused on some or all of the proposed four lung regions [16, 20, 21, 27].

It should also be noted that asthma symptoms, such as CAS, may not necessarily correlate with lung function [1, 7], but they provide different information about the disease state. It has been reported that most asthma patients are not able to precisely correlate their respiratory symptoms to the severity of airway obstruction as measured by pulmonary function tests [46]. In this regard, recording respiratory symptoms, such as CAS, has been reported to contribute to improving the interpretation of spirometry in asthma [2]. Indeed, pioneering studies provided valuable measurements of wheezing for the assessment of certain obstructive pulmonary diseases [48, 49], and showed that the combination of spirometry and RS analysis increases the sensitivity of detection of pulmonary diseases [16].

Hence, in this study, we aimed to assess BDR in terms of acoustic parameters, which may not necessarily coincide with BDR assessed by spirometry, but the combination of the two techniques improves the assessment of BDR in asthma patients.

### Analysis of CAS features

**Fig 9:**
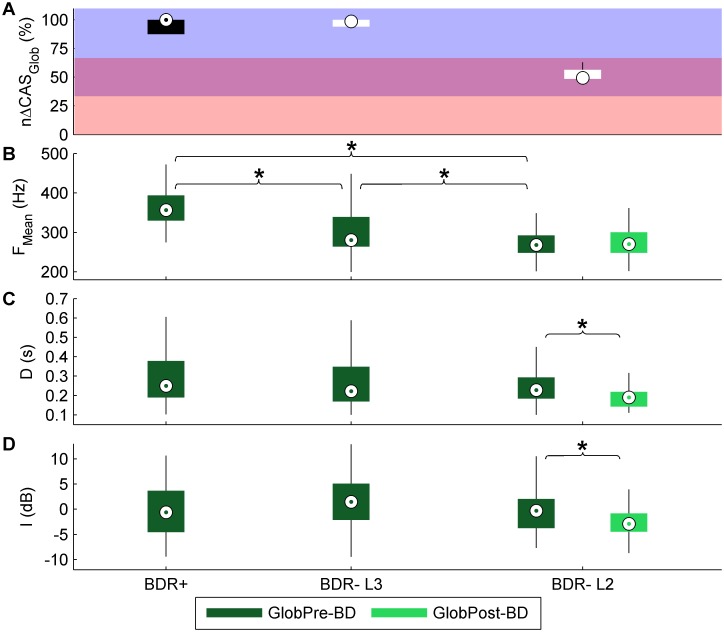
Population CAS features. Change in the number of CAS (*nΔCAS*_*Glob*_) (A). Pink area = low response (L1), purple area = medium response (L2), blue area = high response (L3). Boxplot distributions of the mean frequency (*F*_*Mean*_) (B), duration (*D*) (C), and intensity (*I*) (D) of CAS at baseline (GlobPre-BD) from BDR+ patients (79 CAS components from 5 patients), BDR- patients with a high response (147 CAS components from 6 patients), and BDR- patients with a medium response (59 CAS components from 4 patients). CAS features after bronchodilator administration (GlobPost-BD) are also shown for the BDR- L2 group, since these patients still had an appreciable number of CAS after bronchodilator (27 CAS components from 4 patients). Circles within boxes = medians, boxes = interquartile ranges, whiskers = 1.5*interquartile ranges. Significant differences (p<0.05) are indicated with an asterisk.

Using the Hilbert spectrum allowed CAS to be more accurately characterized with regard to *D*, *F*_*Mean*_, and *I* than using spectrogram, which has been the most widely used technique for CAS analysis, as we previously described [34]. These CAS features highly depend on the airway diameter and the airway wall thickness, stiffness, elastance, and longitudinal tension [32]. In this study, we also aimed to analyze CAS features, since they could help to better characterize and distinguish between patients with different BDR. In particular, we analyzed CAS features in asthma patients who had an appreciable response in the number of CAS, including the 5 BDR+ patients, the 6 BDR- patients with a high response, and the 4 BDR- patients with a medium response (Fig 9).

Normality of the *F*_*Mean*_, *D*, and *I* of CAS from each group was tested using a Lilliefors test. Since not all CAS features had a normal distribution, non-parametric statistical tests were used to analyze the differences in CAS features between groups.

Differences in the *F*_*Mean*_, *D*, and *I* of CAS at baseline were analyzed between the three groups using a Kruskal-Wallis test and multiple post hoc comparisons with the Bonferroni adjustment. Significant differences (p<0.05) were found for the *F*_*Mean*_, with BDR- patients having lower values than BDR+ patients, and BDR- L2 patients having lower values than BDR- L3 patients. Several previous studies reported that the mean frequency of CAS decreased after bronchodilator administration, revealing that CAS containing higher frequencies are associated to a higher degree of airway obstruction [13, 20, 29, 31]. It has also been reported that the pitch of CAS increases with narrower airways, thinner airway walls, or stiffer airway walls [32]. Accordingly, given that airway obstruction was higher (lower FEV_1_ and lower FEV_1_/FVC) in BDR+ patients than in BDR- patients (see Table 1), the *F*_*Mean*_ of BDR+ patients was higher than that of BDR- patients.

Differences in *F*_*Mean*_, *D*, and *I* between CAS at baseline and CAS after bronchodilator administration were analyzed for the BDR- patients with medium response (BDR- L2), who still had an appreciable number of CAS after bronchodilation. In this case, a Wilcoxon rank sum test was used to analyze the difference in each CAS feature. We found significant differences (p<0.05) in *D* and *I*, which were lower after bronchodilation, as reported in previous studies [13, 20, 29, 31]. Bronchodilation reduces the resistance in the airways, which increases the minimum critical flow necessary to generate CAS, reduces the oscillation of airway walls and, as a result, reduces the *D* and *I* of CAS.

Beyond the aforementioned statistical significance of the results, CAS features had high variability in all groups. The differences in CAS features between groups were not strong enough to draw relevant clinical conclusions, which would require a larger population of asthma patients. In any case, it seems that the potential of CAS feature analysis for assessing BDR in terms of acoustic parameters is lower than that of the analysis of the number of CAS.

## Conclusions

This study proposes an innovative, non-invasive, and thorough approach to CAS analysis for assessing BDR. It has been shown that analyzing CAS provides quantitative information that allows the BDR of asthma patients to be assessed in terms of acoustic parameters, beyond those provided by spirometry. Previous attempts to analyze the relationship between BDR and the presence of CAS were limited due to certain methodology issues that have been addressed and overcome in this study. It is noteworthy that we trained and validated a high performance classifier to distinguish CAS from other sounds. This classifier makes our approach to CAS analysis more robust with respect to background noises in the clinical environment, reduces CAS overestimation, and provides a novel tool for assessing patients with obstructive pulmonary diseases in pulmonary function testing laboratories.

The multichannel analysis of CAS allows us to quantify changes in airway obstruction that would not be detected by spirometry. Indeed, we were able to detect appreciable changes in the number of CAS after bronchodilator administration in 10 of the 13 BDR- patients included in this study, revealing some degree of change in airway obstruction. Therefore, together with spirometry, which is a well-established and standard methodology for BDR assessment, the analysis of CAS contributes to improving the stratification of BDR in asthma patients.

### Limitations of the study

The study subjects were within a wide range of airway obstruction levels and BDR, thus enabling the proposed approach to reveal its potential for improving the stratification of BDR levels, particularly among patients with negative BDR. In this regard, we think that the proposed categorization for the response in the number of CAS is reasonable and could be applicable to a large population. Nevertheless, the promising results should be corroborated using a larger number of asthma patients.

Regarding CAS features, the differences between pure low-pitched wheezes and rhonchus, or between stridor and high-pitched wheezes are still unclear, as reported in [10]. Therefore, further studies should address in detail the characterization of different types of CAS. A better understanding of the different types of CAS would help us to improve our acoustic component segmentation and characterization algorithm which, in turn, would contribute to improving the accuracy of our acoustic component classifier.

A key aspect of the proposed methodology is that CAS are not present in all asthma patients and not all CAS are related to asthma. However, when present, CAS are an unequivocal sign of airway obstruction. Accordingly, this approach to CAS analysis is proposed not as a unique technique for assessing BDR, but as a high-sensitive tool for providing useful complementary, objective, and quantitative information about BDR in patients exhibiting CAS, in a simple and non-invasive way. Together with spirometry, this technique has a direct clinical application for improving the stratification of BDR levels and the management of patients with obstructive pulmonary diseases.

## Supporting information

S1 File Study data. Anthropometric and spirometry data of the study subjects (sheet 1). Analysis of the number of CAS of the study subjects in the total flow range (sheet 2). Analysis of the number of CAS of the study subjects in the flow quartiles Q1-Q4 (sheet 3).
(XLSX)

S2 File CAS features. MAT-file containing a 28-by-4 structure array. Each structure includes mean frequency, duration, and intensity of CAS. Rows 1–5: controls, row 6: all controls, rows 7–19: BDR- patients, row 20: all BDR- patients, rows 21–27: BDR+ patients, row 28: all BDR+ patients, column 1: inspiration pre-bronchodilator, column 2: inspiration post-bronchodilator, column 3: expiration pre-bronchodilator, column 4: expiration post-bronchodilator.
(MAT)

S3 File Fig 2 data. MAT-file containing respiratory sound (‘signal’ vector) and flow (‘flow’ vector) signals, starting and ending times of the respiratory phases (‘tins’ and ‘texp’ vectors), and flow quartile labels (‘quartiles’ vector) shown in Fig 2.
(MAT)

S4 File Fig 3 data. MAT-file containing respiratory sound (‘signal*i*’ vectors) and flow (‘flow*i*’ vectors) signals, and segmented acoustic components (‘cas_components*i*’ arrays) shown in Fig 3.
(MAT)

S5 File Fig 4 data. MAT-file containing respiratory sound (‘signal*i*’ vectors) and flow (‘flow*i*’ vectors) signals, and segmented acoustic components (‘cas_components*i*’ arrays) shown in Fig 4.
(MAT)
